# Medical students’ perceptions of factors that Impact their performance in human physiology course: suggestions for improving course presentation

**DOI:** 10.1186/s12909-023-04661-y

**Published:** 2023-09-27

**Authors:** Raed Halalsheh, Amneh Al-Rawashdeh, Eman Rababah

**Affiliations:** 1 College of Applied Medical Sciences, Department of Medical Laboratory Sciences, The Hashemite University, Zarqa, Jordan; 2 College of Education, Department of Educational Administration and Foundations, Yarmouk University, Irbid, Jordan

**Keywords:** Human physiology, Performance evaluation, Medical Applied Health College

## Abstract

**Objectives:**

The study aims to examine students’ perceptions of factors that impact students’ performance in the Human Physiology course at HU’s College of Applied Health Sciences and their suggestions for improvement.

**Method:**

A cross sectional study was conducted between March 2022 and April 2022. A self-administered online questionnaire was distributed to undergraduate students in Physiology courses (online and blended) via Microsoft Teams. Data were analyzed descriptively and inferentially, and thematic analysis was employed based on the most frequent statements for the open-ended question.

**Results:**

In total, 435 students participated in the study. Results indicated that students had high levels of agreement (M = 4.39) regarding faculty teaching style compared to (M = 4.24) towards course content and (M = 3.49) moderate levels towards technological aspects. In terms of the statistically significant differences at (α = 0.05) in students’ perceptions of factors that influence their performance due to the variables (gender, GPA, college, and teaching methods: online or blended), results showed that course content was not affected by any variables. The technological aspects were affected by GPA and gender. In terms of faculty teaching style, it was affected by all variables (GPA, college, and teaching method) except gender. One open-ended question regarding suggested improvements revealed four main themes: assessment and evaluation, technical issues, teaching methods and tools, and Arabic language support.

**Conclusion:**

The study findings recommend greater use of assessment for learning methods and provision of interactive materials to help medical students overcome the challenges that might impact their performance.

## Background

The undergraduate Human Physiology is a required course for all medical students in their senior or sophomore year. Scholarly research over years has identified numerous factors that make learning Physiology challenging. First-year students often find difficulty in grasping theoretical knowledge due to their limited understanding of clinical approaches and some concepts of basic sciences [1–3]. The course requires that previous knowledge of basic concepts from molecular to the complete organism is needed, as well as acquaintance of biology, chemistry, mathematics and physics; such interdisciplinary previous knowledge makes the learning curve more challenging. Students also struggle with rich curricular content and new technical terms [3, 4]. The course is usually offered for large and diverse numbers of students which forces the instructors to teach it in a didactic form (lecture method), whereby students depend on their class notes, teachers’ slides as well as personal efforts for passing the course [3, 4].

Compounding these challenges, the COVID-19 pandemic necessitated significant changes and inflicted worldwide disruptions to education in general and medical education, specifically. Some medical education studies reported lower academic performance due to multiple factors: (1) many students’ academic performance was negatively impacted by the sudden shift to online learning due to limited interaction with peers and instructors [5]. (2) First-semester medical students suffered the most during the pandemic as they lacked face-to-face academic support during the lockdown [5–7]. (3) Medical students were deprived of practical face-to-face training which impacted natural clinical settings and restricted lab time for functional aspects of medicine [8–10]. (4) Technical barriers such as unstable Internet access presented obstacles to stress-free study [11]. (5) There was inadequate preparation, limited resources, and poor technological support [7, 12]. (6) Students also raised concerns about online classes in terms of limited classroom instruction and pedagogy which impacted online assessment and evaluation methods [13].

Therefore, transitioning from face-to-face to online/blended learning requires educators to utilize different pedagogies [14] and for students to self-regulate their knowledge [15]. Regarding pedagogy and teaching strategies, case-based learning (CBL) was found to help students understand the Physiology course matter as it connects theoretical and practical aspects of basic sciences and their relationship to clinical signs, symptoms, and pathophysiological processes [16]. In addition, it was reported that incorporating structured interactive sessions (SIS) in small discussion groups enhances performance by increasing instructor/ student interaction [17]. Also, a combination of instructive lectures, CBL, SIS, and problem-based learning [1] can significantly improve students’ skills and knowledge in the Physiology curriculum.

Whilst all educational institutions worldwide were impacted by the COVID-19 pandemic and its consequences, the Jordanian universities were particularly challenged during the pandemic, especially as most of the Jordanian Universities have limited utilization of E-Learning Platforms and online classes. Therefore, during the beginning of the lockdown, universities were forced to employ different academic and social online platforms (such as Facebook, Messenger, and WhatsApp groups to ensure the educational process proceeded smoothly. At Hashemite University, the undergraduate Human Physiology course was offered face-to-face before the pandemic, and suddenly was switched to entirely online during the pandemic. Major technical issues and problems of accessibility, in addition to the nature of the course, made the online teaching experience challenging. After the pandemic, the College of Applied Medical Sciences at Hashemite University (HU) decided not to offer the physiology course fully face-to-face but rather to offer the Physiology course in two formats; first, an entirely online method and second, a blended approach in response to the National Action Plan for Embedding Online Learning (Full & Blended) policy issued by the Jordanian Higher Education Council and Scientific Research committee, formed in November 2020. This policy aims to enhance the quality of the online and blended teaching and learning process in all Jordanian Higher Education (HE) institutions to meet global standards of teaching and learning.

Accordingly, the undergraduate Human Physiology course was offered in the fully online format for Nursing majors and Medical Engineering students, and a blended format (2 + 1) for the majors of the Medical Laboratory Sciences College. The online class was taught three hours per week, and the lectures were uploaded via the Microsoft Teams platform. In the blended course, instructors meet with students twice on campus (face-to-face) and once online. Both online and blended courses cover the same topics, and the exams are allocated as follows: 30 marks (first exam), 30 marks (second exam ), and 40 marks (final exams). All exams consist of multiple-choice questions, computer-based exams, and are held on campus.

This new format of teaching has been implemented recently, and the current students are considered to be the main recipients of this mode of teaching. Students’ attitudes towards this shift to online and blended classes for large groups of medical students at Hashemite University have not been studied exclusively. Therefore, this study aims to examine students’ perceptions of factors that impact students’ performance in the Human Physiology course at HU’s College of Applied Health Sciences and their suggestions for improvement.

## Methods

### Study design and setting

This cross-sectional study was conducted using an online questionnaire in the second semester of the academic year (2021/2022) with voluntary participation from students studying the Physiology course at Hashemite University. The Institutional Review Board (IRB) of the Hashemite University approved the study (IRB No:2,200,218).

### Profile of the research subjects

Hashemite University (HU) offers the undergraduate Human Physiology course in six colleges: Medicine, Nursing, Dentistry, Pharmacy, Biomedical Engineering, and Applied Medical Sciences. This study examines the Human Physiology course offered by the College of Applied Medical Science through the Medical Laboratory Sciences department as the focus of the course is different in other colleges in the same university. This course is a required course for all programs in the Nursing college and the Biomedical Engineering department from the college of Engineering, and all majors in the Applied Medical Sciences. The theoretical course focuses on the human body’s essential functions, including cell, muscles, nervous, cardiovascular, urinary, reproduction, and immune systems, and the mechanisms involved in the physiological processes. The course aims to introduce students to basic concepts and principles of human physiology and relationships between various body systems and provide the scientific foundation for medicine and all other professions related to human health and physical performance.

The class enrolls an average of 100–150 first-year or sophomore students per section and has a general biology course as a prerequisite. The student body of this course varies in term of majors, previous knowledge, and skills in English language. The course materials, slides, and exams are written in English as it is the required language for the Medical Sciences at Hashemite University and in Jordan. Most professors try to present the course terminologies in Arabic as well as in English. In addition, the Applied Medical Sciences majors must take an Anatomy course before embarking upon the Physiology course. Nursing students take both Physiology and Anatomy courses in the same semester. The Applied Medical Sciences students (Medical Laboratory Sciences, Physiotherapy, Occupational Therapy, Clinical Nutrition, and Clinical Imaging) take the one-credit Human Physiology laboratory to parallel the theoretical course. In contrast, the remaining majors must take the one-credit Human Physiology laboratory.

### Questionnaire validation, reliability, and pilot testing

The initial questionnaire was designed and drafted in Arabic, the native language of Jordan. The content validity of the questionnaire was verified by six independent scientists in the field of Physiology who gave their feedback in terms of clarity of meaning, linguistic formulation, and the relevance of the questionnaire statements to the objectives of the study. Based on the reviewers’ comments, six statements were re-rewritten for language clarity.

Statistical reliability and category validity were verified through the Cronbach alpha coefficient. Preferred coefficient values are above 0.70 (acceptable to be above 60). Table 1 below shows the Cronbach alpha coefficient values for each category:

**Table 1:** The Cronbach alpha coefficient

Factor	Cronbach α
**Faculty teaching style**	0.866
**Course content**	0.668
**Technological aspects**	0.734

### Analytical methods and tools

A cross sectional approach was adopted, and an online questionnaire collected students’ perceptions towards factors that impact students’ performance in the Human Physiology course and their suggestions for improvement. SPSS (V.26) provided initial data screening, descriptive analysis, Exploratory Factor Analysis (EFA), and significant differences using Independent Samples T-Test and One-Way Analysis of Variance (ANOVA). AMOS (V.23) provided Confirmatory Factor Analysis (CFA). EFA was applied to extract measurement factors, hence factor structure was placed initially in EFA. Subsequently, CFA verified the factor’s design. Finally, thematic analysis was employed for the open-ended question based on the most frequent statements.

### Data collection, screening, and preliminary analysis

After drafting the questionnaire and feedback-based revision, the questionnaire was created using the “Google Forms” online survey platform (Google LLC., Mountain View, CA). It was then distributed directly to all students taking the Physiology courses (online and blended) using Microsoft Teams. The questionnaire’s introduction stated that no personal information or identification of respondents was required. In addition, students were informed that data would be confidential and professionally handled according to scientific research standards and ethics and that they could withdraw their answers at any time during the study without any explanation.

The questionnaire consisted of 11 statements in three categories: faculty teaching style, technological aspects, course content, and there was one open-ended question. Data collection occurred between March 2022 and April 2022, and 447 responses were received. An Excel sheet was exported to conduct statistical analysis. First, the validity of gathered responses was checked by examining standard deviation (std.) values for Likert-based statements; 12 replies scored very low std. Values showing regular patterns, indicating invalid responses, were dropped from the sample. Further, plot-dot diagrams were examined, and no significant outliers were found in the dataset. The final model presented for further analysis comprised 435 responses.

## Results

### Demographic characteristics of medical students

After the questionnaire was distributed to participants towards the end of the second semester at Hashemite University, (435) responded. Participants’ demographics are presented in Table 2, which shows that out of (435 participants) 47.8% were male and 52.2% were female students. Most of the participants have a good GPA (40.0). The vast majority of participants were from the College of Nursing (78.4%). A total of (79.1%) of participants were enrolled in an online class. The demographic characteristics of the participants who completed the questionnaires are shown in Table 1 which presents a summary of students’ characteristics.

**Table 2:** Students’ characteristics summary (n = 435)

Characteristic	Subset	Count	%
Gender	Male	208	47.8%
	Female	227	52.2%
	*Total*	*435*	*100%*
GPA	Fair	46	10.6%
	Good	174	40.0%
	Very good	150	34.5%
	Excellent	65	14.9%
	*Total*	*435*	*100%*
College	College of Applied Medical Sciences	63	14.5%
	College of Nursing	341	78.4%
	College of Engineering	31	7.1%
	*Total*	*435*	*100%*
Course delivery	Blended	91	20.9%
	Online	344	79.1%
	*Total*	*435*	*100%*

The answer to the first question about the factors impacting students’ performance in Human Physiology courses at the College of Applied Health Sciences at Hashemite University is a descriptive analysis including mean and standard deviation (std). The descriptive analysis provided aggregate students’ perceptions levels towards each proposed factor. The scale proposed by Sekaran and Bougie [18] was used to interpret mean values: a low level of agreement fell within (1–2.339), a moderate level fell within (2.34–3.66), and a high level fell within (3.67–5.00). Table 3 presents mean and standard deviation values for students’ perceptions of factors impacting their performance in the Physiology course.

**Table 3:** Factors that Impact Students’ Performance

No.	Order	Statement	Mean	Std.	Level
*Faculty teaching style*					
1	2	Uses relevant real-life examples to clarify the topic	4.43	0.84	High
2	1	Engages with students effectively	4.64	0.77	High
3	3	Appreciates students’ knowledge and experience	4.40	0.94	High
4	4	Adapts teaching methods in relation to students’ needs	4.38	0.91	High
5	5	Makes learning process pleasurable	4.11	1.10	High
*Overall mean*			*4.39*		*High*
*Technological aspects*					
1	2	I own devices that enable me to succeed in class	3.44	1.12	Moderate
2	1	I have enough technical skills to use the online platform	3.71	1.11	High
3	3	I have good Internet access	3.32	1.12	Moderate
*Overall mean*			*3.49*		*Moderate*
Course content					
1	2	The physiology course presents a large volume of information	4.34	0.83	High
2	3	The course requires prior knowledge of biology	4.00	0.97	High
3	1	The course requires knowledge of new terms in anatomy and physiology	4.40	0.88	High
*Overall mean*			*4.24*		*High*

Table 3 shows that respondents had high levels of agreement (M = 4.39) regarding faculty teaching style. Students perceived this to be an essential determinant of academic performance. There were high levels of understanding for all aspects of this determination, showing HU’s Physiology instructors meet an excellent standard of teaching. However, the element suggesting “*Makes learning process pleasurable”* scored a standard deviation value above (1), showing student disagreement.

Results revealed a high level of agreement towards all statements of the course content determinant, with mean values ranging between (4.40) to (4.00) (overall mean value of (M = 4.24). Std. Values were below (1), showing agreement among students. Respondents returned overall moderate perceptions (M = 3.49) towards technological aspects, with all std. values below (1). This indicates that some students require help in acquiring the necessary technical means.

The second question addresses the significant differences in perception levels of proposed determinants due to characteristics: (Q2: Are there any statistically significant differences at (α = 0.05) group in the students’ assessments for factors that influence their performance in the Physiology course due to the variables of gender, GPA, college, and teaching methods (online, blended). The Independent Sample T-Test was applied to test for gender and delivery variables. In addition, the ANOVA test was used to test for GPA and college variables. These parametric tests are valid considering that preliminary analysis, based on skewness and kurtosis measures, revealed no data abnormality issues. Table 4 summarizes the results.

**Table 4:** Difference in Perceptions to Proposed Determinants

Determinant	F	Sig.	Sig. group
*College*			
Faculty teaching style	16.905	0.000*	College of Nursing -- College of Engineering
Technological aspects	2.483	0.085	--
Course content	0.553	0.575	--
*GPA*			
Faculty teaching style	10.782	0.000*	Good -- Very good -- Excellent
Technological aspects	7.233	0.000*	Very good -- Excellent
Course content	1.226	0.300	--
**Determinant**	**T**	**Sig.**	**Sig. group**
*Gender*			
Faculty teaching style	-1.728	0.085	**--**
Technological aspects	-2.183	0.030*	**Female**
Course content	-0.942	0.347	**--**
*Delivery method*			
Faculty teaching style	-6.113	0.000*	**Online**
Technological aspects	-0.864	0.388	**--**
Course content	-0.768	0.443	**--**

The ANOVA tests reported non-significant differences according to college in technological aspects and course content perception levels. Test values were (F = 2.483) and (F = 0.553), respectively, with P-values above (0.05). Students at all colleges reported the same levels of perceptions toward these determinants. Meanwhile, ANOVA was significant (F = 16.905) for differences in faculty teaching style showing substantial differences. Scheffe’s post-test reported that students from the Colleges of Nursing and Engineering reported higher perceptions of faculty teaching style than students in the Applied Medical Sciences College. ANOVA showed non-significant differences in students’ perceptions of course content due to their GPA. A test value of (F = 1.226) shows that students share similar levels of perception toward course content, regardless of GPA. Conversely, ANOVA tests reported significant differences due to GPA in faculty teaching perceptions (F = 10.782) and technological aspects perceptions (F = 7.233). In addition, Scheffe’s post-test reported that students with a ‘Good,’ ‘Very good,’ or ‘Excellent’ GPA reported higher perceptions than students with a ‘Fair’ GPA. Also, students with a ‘Very good’ or ‘Excellent’ GPA reported higher levels of technological aspects perceptions than students with a ‘Fair’ or ‘Good’ GPA.

T-Test reported non-significant differences in perceptions regarding faculty teaching style and course content due to gender. Values were respectively (T= -1.728) and (T= -0.942) with P-Value above (0.05), showing that both male and female students share similar perceptions towards teaching style and course content determinants. Conversely, the test reported significant differences in technological aspects due to gender, recording (T= -2.183) with a P-value less than (0.05). A comparison of mean values showed that females have higher perceptions than males regarding technological aspect determinants.

T-Tests reported non-significant differences in technological aspects (T= -0.864) and course content (T= -0.768). Perception levels due to the delivery method gave a P-Value above (0.05). Participants reported comparable perceptions of these two determinants regardless of the delivery method. The test showed significant differences in faculty teaching style due to the delivery method, as the test value was (T= -6.113). Students receiving online courses reported higher levels of perceptions toward faculty teaching style compared to students participating in a blended approach.

The open-ended question relating to respondents’ suggestions for factors practices that could improve students’ performance in the Physiology course revealed four main themes that emerged from the data: (1) assessment and evaluation theme in which the participants referred to how faculty can utilize assessment to encourage students to learn how to retain knowledge for the exams, (2) technical issues referred to how the university technological services can support students’ learning and ensure accessibility, (3) teaching methods and tools referred to teaching practices and aids that can make the learning process more engaging and interactive for students, and (4) Arabic language support referred to providing supportive materials for terminology in physiology in Arabic. Suggested factors are shown in Table 5.

**Table 5:** Suggested Factors to Improve Students’ Performance –

Suggestions	F
Assessment & Evaluation	
Frequent short exams/ quizzes at least after each chapter to ensure that the information is understood and communicated and allow students to improve course mark	82
Count the highest scores of quizzes given during the semester	49
Do more revision before exams/ review each chapter	22
Assign quizzes/ homework to groups so that each group explains a quiz/ homework to classmates	19
Allocate marks to encourage student participation	34
Marks are limited to exams only. Other mechanisms must be adopted to measure the student’s understanding, not just memorization.	12
Technical Issues	
Solve the problems of the Teams application, especially during the 5 o’clock lecture because numerous lectures coincide, putting pressure on university servers.	80
The university must provide students with an alternative interactive application to the Teams application because of its numerous problems.	75
Consider students’ technical capabilities when setting up online classes (Internet connection, laptop, tablet, etc.)	107
More focus on quarterly work (such as research, reports, and assignments).	39
The instructor should ask 5 to 6 random students questions about the previous lecture to enhance students’ commitment to studying each class.	86
Teaching Methods and Tools	
Conduct periodic face-to-face lectures on campus to review the previous lectures. Students study regularly and don’t forget information quickly when studying is interactive.	41
Instructor should allow cameras and microphones to encourage interactive discussion and activities during lectures.	104
Use modern teaching aids that present course content in attractive ways / provide better video/audio quality/3D video content	108
Put what has been taught into practice/ practical laboratories	22
Due to intensive course content, instructors should provide detailed summaries of material highlights / explanations of medical terms	50
Instructor should use real life examples to explain concepts	12
Arabic Language Support	
Due to English language issues/ lack of Arabic language references, instructor or department should provide students with explanations of material in Arabic.	76

## Discussion

This study examines students’ perceptions of factors impacting their performance in the Human Physiology course at the College of Applied Health Sciences at Hashemite University as we switched to offering the course in online and blended format instead of face-to-face format. Results indicated that students had high levels of agreement (M = 4.39) regarding faculty teaching style compared with (M = 4.24) in respect of course content and moderate levels (M = 3.49) in relation to technological aspects. These results could be explained by the fact that freshman and sophomore students view and expect instructors to be the primary disseminator of knowledge and experts who can ease the difficulty of the Physiology course; this is especially given the fact that their previous K-12 education level and approach would have been mainly a teacher centered approach based on memorization and retaining information for the exam. Therefore, when they come to the university setting, they depend heavily on the instructor to clarify concepts, and connect theoretical knowledge to real-life examples. In addition to that, freshman and sophomore students enter university with a minimum basic knowledge in biology, chemistry, and mathematics which makes courses like physiology very challenging. This result aligns with other studies reporting that teacher didactics were also seen as influential in teaching and learning Physiology [3]. Kaddam and Elnimeiri’s study [19] asserted that Sudanese students considered direct teaching influential and felt that their absence could harm their performance. Salisu et al. [20] reported that Nigerian students believed that improving pedagogical strategies and changing teachers’ attitudes towards students could improve their performance in the discipline. Scholarly research has claimed that teachers are one of the most critical school-based influences on student academic performance [21, 22], accounting for up to 30% of the variance in student achievement [23]. Similar results in different educational contexts indicate that instructors are vital in the learning process, regardless of the teaching platform.

Course content is the second interrelated factor that impacts students’ performance (M = 4.24). This result aligns with other research which has reported that the nature of the Physiology course affects students’ learning due to its complexity and interconnectivity to other disciplines [1–4]. This result also relates to the vital role of the instructor in organizing and managing the heavy content in a way that helps students learn the materials and rationalize the connectivity of human physiology to different disciplines. Both results could also explain the moderate levels of impact (M = 3.49) towards technological aspects because, in our context, we make the class materials (PowerPoint supporting materials) available for the students on the platforms, and we keep the recorded materials during the semester; therefore, students do not feel challenged to stay connected during the class as they can go back to the materials and classroom discussion whenever they wish.

In terms of the statistically significant differences at (α = 0.05) in students’ perceptions towards factors that influence their performance due to the variables (gender, GPA, college, and teaching methods (online, blended), results showed that course content was not affected by any variables. The fact that course content was not affected by any variables can be attributed to the fact that the Physiology course is challenging for students regardless of their GPA, college, gender, or teaching method. Technological aspects were affected by GPA (students with a ‘Very good’ or ‘Excellent’ GPA reported higher levels of technical aspects perceptions than students with a ‘Fair’ or ‘Good’ GPA) and gender (Female). This result can be attributed to the fact that students with high GPAs prefer to discuss and interact with teachers through synchronous activity rather than asynchronous. Regarding gender (female), a high level of perceptions toward technological aspects can be explained by the fact that female medical students are perceived as higher achievers than males within Jordanian society, making them concerned about connectivity and the internet as it might affect their performance negatively.

In terms of faculty teaching style, it was affected by all variables (GPA, college, and teaching method) except gender. The Colleges of Nursing and Engineering reported higher perceptions of faculty teaching style than students in the Applied Medical Sciences College. This can be explained by the fact that the course plan for non-Applied Medical Students in Sciences requires them to register for the physiology course within the first academic year compared to Applied Medical students who can take the course during their second year. Students receiving online courses reported higher levels of perceptions toward faculty teaching style compared to students participating in a blended approach. This can be attributed to the fact that the combined students meet physically with the instructors weekly, giving them more opportunities to ask questions during or after the class. Students with a ‘Good,’ ‘Very good,’ or ‘Excellent’ GPA reported higher levels of perceptions toward faculty teaching style than students with a ‘Fair’ GPA. This can be explained by the fact that Fair GPA students might not be aware that the instructor’s teaching style will help them to perform better in the class.

With respect to students’ suggestions regarding factors that could impact their performance in online and blended courses, four major themes emerged based on the frequencies. In the first theme, teaching methods and tools, students highlighted the role of faculty teaching methods and tools as a major factor that contributes to enhance their performance. a total of (108) students suggested, “using modern teaching aids the present course content in attractive ways / provide better video/audio quality/3D video content”. Students in the Physiology course encounter hurdles in concretizing abstract concepts in physiology and visualizing different physiological processes, integrating relative concepts, and understanding the relationship between various body systems. Thus, they think that instructors must enrich the learning process and effectively use technology and supporting learning materials to connect the theoretical concepts visually. This result aligns with the previous result in the questionnaire that participants had high levels of agreement (M = 4.39) towards faculty teaching style. Bhalli et al. [24] study reported that medical students perceive interactive academic classes to benefit their learning. Wynter et al. [25] found that 92% of students rely on educational videos for learning new material or reviewing learned concepts.

In the second theme, Assessment & Evaluation, students suggested that instructors need to utilize evaluation and assessment efficiently to enhance learning and improve student performance. A total of (82) respondents indicated that instructors must provide “frequent short exams/ quizzes at least after each chapter to ensure that the information is understood and communicated and allow students to improve course mark.” This suggestion can explain that whilst some instructors might try to assess students’ learning, this was not considered to be in an efficient way that supports students’ knowledge and enhances performance. The Human Physiology course is supposed to be a designed course in which all building units/chapters support each other so that students are encased within a supportive learning system. To help students learn and perform well in the course, the instructor needs to employ a system of assessment for learning. It is an approach to teaching and learning that aims to (1) collect information about student learning before, during, and at, or near the end of, a period of instruction, using a variety of assessment strategies and tools; (2) employ different assessment tools to inform instruction, and guide students to monitor their progress towards achieving their learning goals. This assessment approach is needed in the medical field, especially given that medical students have demanding courses that require them to self-regulate their time, and most of the studying for the exam takes place at the last minute. In response, students think that applying formative assessment (quizzes, chapter revision, and classroom discussion) during the semester could help them better regulate their time with the material, ultimately leading to better performance. Bickerdike et al. [26] reported that the proportions of students in Irish medical schools utilizing a “cramming” strategy (most class materials before evaluations) or a “consistent” approach (constant throughout the academic year) were 47.1% and 49.7%, respectively, with students who adopted “cramming” habits tending to earn poorer scores.

In the third theme, students referred to technical issues that must be addressed in the university setting. A total of (107) students suggested that the university needs to “consider students’ technical capabilities (Internet connection, laptop, tablet, etc.)”. Students’ suggestions align with previous research [7, 12] about the connectivity issues in online and blended classes and how it relates to students’ learning. Finally, in the fourth theme ‘language barriers’, (67) students highlighted the universities and departmental efforts to “provide students with an explanation of material in Arabic.” At Hashemite University, the textbook, materials, and exams are presented for all medical students in English. Thus, students feel challenged to grasp the concepts in the course, especially the majority of the students who come from public school and have been teaching science classes in Arabic. This suggestion is important in our context as it raises questions about our efforts as a faculty to create supporting materials in Arabic to ease the difficulty of courses such as Physiology.

Understanding how multiple factors impact students’ performance and eliciting students’ suggestions for improving the teaching and learning of the course could further an understanding of how best to serve the students. This is important as the course functions as a foundational base for other medical courses, practical and laboratory experiences, and contributes to preparing competent graduates to join the medical workforce in Jordan.

## Conclusion

This study emphasizes that in this educational context, regardless of the teaching platform, face-to-face or online /blended instruction is the main factor that impacts students’ performance in physiology class. It can be concluded that teaching physiology requires not only a thorough understanding of the subject matter, but also the ability to convey complex concepts in a way that is engaging and accessible to all students. One key aspect of teaching biology effectively is the ability to break down complex concepts into manageable pieces for students. This involves using clear, concise language for students in their mother language and providing plenty of examples and illustrations to help students grasp the material. It also involves using a variety of teaching methods, such as hands-on activities, demonstrations, and visual aids, to help students better understand and retain the information.

This study is important as it is one of the few studies in the medical field in Jordan that tries to examine factors that impact students’ performance. Trying to reflect our educational practices, what we are doing, and what can be done to help students learn better and spark their curiosity about the subject is vital. This study may suggest that an instructor’s knowledge in utilizing pedagogy and assessment can facilitate students learning, which asserts that how we teach and assess is much more important than what we teach [27].

This study sheds light on an important aspect about factors that impact students’ performance in the Physiology course at Hashemite University, which is one public university in Jordan, and one medical college. Nevertheless, it has raised some generalizable issues and further studies could be conducted to examine students’ perceptions in other medical universities and colleges globally, as well as employing other research methods such as interviewing students for more detailed insights.

## Data Availability

The data supporting this study’s findings are available on request from the corresponding author, Dr. Raed Halalsheh.
